# A blend of medium-chain fatty acids, butyrate, organic acids, and a phenolic compound accelerates microbial maturation in newly weaned piglets

**DOI:** 10.1371/journal.pone.0289214

**Published:** 2023-07-28

**Authors:** Natalie E. Diether, Tetske G. Hulshof, Benjamin P. Willing, Theo A. T. G. van Kempen

**Affiliations:** 1 Department of Agricultural, Food & Nutritional Science, University of Alberta, Edmonton, Alberta, Canada; 2 Trouw Nutrition, Boxmeer, The Netherlands; 3 North Carolina State University, Raleigh, North Carolina, United States of America; University of Life Sciences in Lublin, POLAND

## Abstract

Inclusion of additive blends is a common dietary strategy to manage post-weaning diarrhea and performance in piglets. However, there is limited mechanistic data on how these additives improve outcomes during this period. To evaluate the effects of Presan FX (MCOA) on the intestinal microbiota and metabolome, diets with or without 0.2% MCOA were compared. Pigs fed MCOA showed improved whole-body metabolism 7 days post-weaning, with decreased (*P* < 0.05) creatine, creatinine and β-hydroxybutyrate. Alterations in bile-associated metabolites and cholic acid were also observed at the same time-point (*P* < 0.05), suggesting MCOA increased bile acid production and secretion. Increased cholic acid was accompanied by increased tryptophan metabolites including indole-3-propionic acid (IPA) in systemic circulation (*P* = 0.004). An accompanying tendency toward increased *Lactobacillus* sp. in the small intestine was observed (*P* = 0.05). Many lactobacilli have bile acid tolerance mechanisms and contribute to production of IPA, suggesting increased bile acid production resulted in increased abundance of lactobacilli capable of tryptophan fermentation. Tryptophan metabolism is associated with the mature pig microbiota and many tryptophan metabolites such as IPA are considered beneficial to gut barrier function. In conclusion, MCOA may help maintain tissue metabolism and aid in microbiota re-assembly through bile acid production and secretion.

## Introduction

In pig production, weaning is associated with many changes in gastrointestinal tract structure, function, and inflammatory status, which increase the risk of post-weaning diarrhea [1]. These physiological changes include decreased digestive and absorptive function resulting from low feed-intake [2], and are exacerbated by changes in dietary composition and the resulting need for alterations in dietary enzyme secretion [3–5]. Concurrently, changes in gut-barrier function, elevated cortisol, and immune cell recruitment result in altered inflammatory status [6–8]. This altered immune and secretory profile alongside alterations in the gastrointestinal microbiota have important implications for pathogen expansion and post-weaning diarrhea [1, 9–11]. One strategy to improve pig outcomes across the weaning transition is through dietary additives [9, 12]. Additive blends, such as those containing medium-chain fatty acids (MCFAs), phenolic compounds, and organic acids, are commonly used during the weaning transition; their effects on the gut microbiota are thought to reduce the risk of gastrointestinal pathogen colonization and adverse outcomes during this vulnerable period in pig production [10, 13].

Medium-chain fatty acids exert antimicrobial effects through disruption of phospholipid membranes [14], and have been shown to suppress gastric and intestinal pathogens, including *Escherichia coli* and *Clostridium perfringens* [14–16]. Short-chain organic acids are often used following weaning to reduce gut pH and pathogen colonization in the gastrointestinal tract [17, 18]. Phenolic compounds have been found to have multiple benefits including reducing oxidative stress, improving lipid metabolism, controlling pathogen colonization, and improving gut barrier function, while increasing the abundance of many core constituents of the microbiota [13, 19, 20]. Supplemental butyrate provides an energy source for intestinal epithelial cells, conferring benefits to gut barrier function while reducing inflammatory cytokines and diarrhea incidence in weaned pigs [21–24].

Synergistic effects of blends of MCFAs and short-chain organic acids have also been demonstrated compared to MCFAs or organic acids fed alone [25]. Previous studies have shown a positive effect of a blend of both MCFAs and organic acids on commensal ileal microbes during pathogen challenge [26], as well as on diarrhea, immune function, and fecal microbiota under non-challenge conditions [27]. Inclusion of these blends in weanling pig diets have also been shown to improve growth, feed efficiency, and nutrient digestibility [28, 29].

While there are many studies demonstrating the synergistic effects of these ingredients in weanling pig diets, the mechanisms by which they affect microbial and host metabolism remain to be examined. Further understanding of how these additives alter microbe-host metabolic networks is critical to their optimal implementation, and to understanding variability in response to these additives under different production conditions. To gain an increased understanding of how a blend of MCFAs, phenolic compounds, butyrate, and organic acids (MCOA) impact microbial and host metabolism, we performed an in-depth characterization of the metabolites in two intestinal sites, portal plasma, and systemic plasma at multiple time points through the weaning transition. These results were combined with microbiota sequencing analysis from the same intestinal sites to explore how modulation of microbiota altered metabolite pools compared to pigs fed a control diet. This approach provided novel insights into the possible mechanisms through which MCOA improve performance outcomes.

## Materials and methods

The study was approved by the Dutch Animal Ethics Committee (AVD2040020184665) and carried out at the Swine Research Centre of Trouw Nutrition R&D (Sint Anthonis, the Netherlands).

### Animal performance data

At weaning (22–25 days of age with average body weight 7.7 kg ± 0.7), a total of 108 pigs (Hypor Maxter × Hypor Libra) were selected from 20 litters of origin and assigned to one of two treatment groups (Table 1), those receiving the control diet (CON) and those receiving a control diet with the addition of a blend of medium chain fatty acids, organic acids, slow release C12, target release butyrate and a phenolic compound (MCOA) (Presan FX, Trouw Nutrition, Amersfoort, NL) supplemented at 0.2% on top of the control diet. Diets were isoenergetic and isonitrogenous (Table 2). Groups were balanced for body weight, colostrum intake, and sex. Pigs were housed in one of six pens with 18 pigs/pen according to treatment and individual feed and water intake data were collected using electronic feeding and water stations (Schauer Agrotronic, GmbH, Austria). Body weights were collected at 2 days prior to weaning, and on days 0, 2, 4, 6, 13 and 27 post-weaning as well as at time of euthanasia. Growth performance data were compared using the PROC MIXED procedure in SAS (SAS Institute Inc., Cary, NC) with treatment as a fixed effect and pen as a random effect.

**Table 1:** Diet formulation of basal diet.

Ingredient	%^1^
Barley	12.3
Wheat	35.0
Maize	10.2
Wheat bran	2.0
Soybean meal 48	20.0
Potato protein	2.5
DL-Methionine 99%	0.18
L-Lysine HCl 98%	0.51
L-Threonine 98%	0.16
L-Tryptophan 98%	0.03
L-Valine 96.5%	0.08
Na Bicarbonate	0.26
Ca Carbonate	0.73
Monocalcium phosphate	0.75
Salt (NaCl)	0.44
Premix	1.0
Lactose	6.5
Sugar	2.5
Soybean oil	4.7
Vitamins and other^2^^,^^3^	0.2

^1^ Ingredients listed constituted the control diet, while the MCOA diet was identical except for the addition of 0.2% MCOA (Presan FX, Trouw Nutrition, Amersfoort, NL) on top.

^2^ Supplied per kilogram of complete diet: Vitamin A, 8000 IU; Vitamin D3, 2000 IU; Vitamin E, 30 IU; Vitamin K3-menadione, 1.5 mg; Vitamin B12, 0.03 mg; Thiamine, 1.00 mg; Niacin, 20 mg; Riboflavin, 4 mg; Pantothenate, 13 mg; Folic acid, 0.30 mg; Pyridoxine, 1.0 mg; Iron sulphate, 100 mg; Zinc sulphate, 100 mg; Magnesium oxide, 30 mg; Copper sulphate, 20 mg; Copper chelate, 70 mg; Sodium selenite, 0.30 mg; Iodine, 1 mg.

^3^ Includes vitamin premix described above as well as 0.04% phytase, 0.12% vitamin E, and 0.02% vitamin B4 (choline).

**Table 2:** Nutrient analysis of experimental diets.

Nutrient^1^	CON	MCOA
Net Energy, kCal/kg^2^	2550	2550
Moisture %	10.7	10.7
Crude protein, %	19	19.1
Crude fat, %	6.4	6.5
Crude fibre, %	2.7	2.7
Ash, %	4.6	4.6
Na, %	0.25	0.26
K, %	0.75	0.76
Mg, %	0.2	0.21
Ca, %	0.67	0.67
P, %	0.45	0.46
Zn, mg/kg	117	108
Sorbic acid, mg/kg^3^^aa3^	<1	130

^1^ Diets were formulated using a wheat, barley, maize, and soybean meal base to meet or exceed all nutrient requirements.

^2^ Net Energy calculated using Bestmix® (Adifo N.V., Maldegem, Belgium) formulation software.

^3^ Analysis of sorbic acid content was performed in order to confirm the addition of MCOA

### Nutrient analysis

Nutrient levels were determined using standard analytical methods. Moisture was determined according to method 930.15 [30], and nitrogen was determined by the combustion method (method 990.03; LECO FP 528 MI, USA) using the LECO Nitrogen analyzer and crude protein calculated as nitrogen × 6.25. The crude fat was determined as an extraction method 920.39 according to AOAC [30]. The ash content of the diets was measured according to method 942.05 [30]. Mineral analyses were completed according to NEN-EN 11510(EN) specifications (2017). This method uses the inductively coupled plasma atomic emission spectroscopy (ICP-AES) method to determine minerals in animal feed after dry ashing. Sorbic acid was determined using the method described by Canale et al. [31].

### Sample collection

On the day prior to weaning, 10 piglets were selected for sample collection based on individual body weight, colostrum intake, and sex. This group was used as reference group that had not been exposed to weaning stress. On D 3, 5, 7, and 14 post-weaning, a subset of pigs was selected for sample collection based on representative feed intake for their treatment group and balanced for sex (n = 10/group/day). Samples from CON animals only were also collected on D28 (n = 10). Prior to euthanasia, piglets were sedated using a mixture of Zoletil (250 mg zolazepam and 250 mg tiletamine) and 20 mL Sedanum (20 mg xylazine / mL) at 1 ml per 10 kg BW. Thereafter, a blood sample was taken from the jugular vein for metabolomics analysis. Blood was stored on ice immediately upon collection, and plasma was isolated by centrifugation (10 minutes at 2000×G at 4°C) within 2 hours after blood collection. Following blood sampling, pigs were euthanized, by an intra-cardiac injection with 40% barbiturate pentobarbital. The abdominal cavity was opened, and a blood sample was taken from the portal vein. The gastrointestinal tract from duodenum to the rectum was removed and clamps were placed at the end of the ileum and at the cecum to avoid mixing of contents. Contents from the complete length of the small intestine were collected and homogenized, while in the large intestine, contents of the mid-colon were sampled. All samples were snap frozen on dry ice and stored at -80°C until further analysis.

### Metabolomics

Plasma and digesta samples were processed by the Center for Proteomics and Metabolomics (Leiden University Medical Centre, Leiden, the Netherlands) and submitted to untargeted LC-MS/MS with a positive and negative ionization phase. Samples were first homogenized, and protein precipitation performed [32]. Metabolomic analysis was performed using an LC-MS/MS based SWATH method with a Shimadzu Nexera X2 system (Shimadzu, Hertogenbosch, The Netherlands). The MS was a Sciex TripleTOF 6600 (AB Sciex Netherlands B.V., Nieuwerkerk aan den IJssel, NL) operated in positive and negative ESI mode. Peaks were identified using data independent acquisition (SWATH) using the public metabolite (VS12) database to align and identify metabolites [33].

Following peak identification, metabolite differences for each day and location were analyzed using Metaboanalyst 5.0. Metabolite intensities were first normalized using quantile normalization and range scaling. Following processing, metabolome differences were visualized using PCA and ANOSIM analysis on Bray-Curtis distances performed to compare differences in metabolomes. Differences in individual metabolites were assessed using FDR adjusted t-tests where significance was determined as *P* < 0.05 and trends at *P* < 0.10. Overrepresentation analysis was performed on jugular metabolite samples using the list of significantly different metabolites to identify metabolic pathway differences between groups at each timepoint.

### Microbiota analysis

RNA extraction was performed on lysed cells (MagNA Lyser; Roche, Burges Hill, UK) using as the MO BIO RNA isolation kit (Carlsbad, CA, USA) without the use of β-mercaptoethanol and DNase in steps as previously described (15). Amplicon libraries of the V3-V4 region of 16S rRNA were constructed following the Illumina protocol with modifications to reduce PCR bias [34].

Sequencing was performed on the Illumina MiSeq platform with a read length of 300bp. Raw sequence data was processed and annotated in Mothur (v 1.39) where reads were merged, filtered for quality, and aligned using the SILVA (NR-123) database [35]. Aligned reads were further filtered to exclude chimeras and clustered into OTUs using VSEARCH with a cut-off of 97% identity [36]. Sequence data was uploaded to the NCBI sequence read archive (Project ID: PRJNA928417). Processed OTU tables and phylogenetic trees were then imported into R where the effects of MCOA on both alpha and beta diversity were analyzed on each day using the phyloseq package [37]. Alpha diversity was examined using both Chao1 and Shannon indices and compared using a Wilcoxon ranked-sum test. Differences in beta diversity were compared using the adonis2 function (PERMANOVA) in the R package vegan on Bray-Curtis and UniFrac matrices. Differentially abundant taxa were examined using LEfSe analysis [38].

Due to the high number of low abundance and low incidence OTUs in the small intestine of pigs sampled on D3, 5 and 7, a successional core was identified from the microbiota of D14 pigs and used for comparisons of core microbiota assembly in the small intestine at earlier timepoints [39, 40]. Due to the disruption of weaning, core microbes were defined as those present in > 60% of D14 individuals with > 5% relative abundance. These OTUs were then compared between groups at D7 using a Wilcoxon ranked-sum test. To further assess the relationship between metabolites and core microbes in the small intestine, regularised Canonical Correlation Analysis (rCCA) was performed using a cross-validation (ridge) method for regularisation in the R package mixOmics [41, 42]. Results were visualised using a relevance network plot with a correlation threshold of 0.6 and a cluster image map showing all correlated relationships between microbes and metabolites identified using rCCA [43].

## Results

### Pig performance

**Fig 1:**
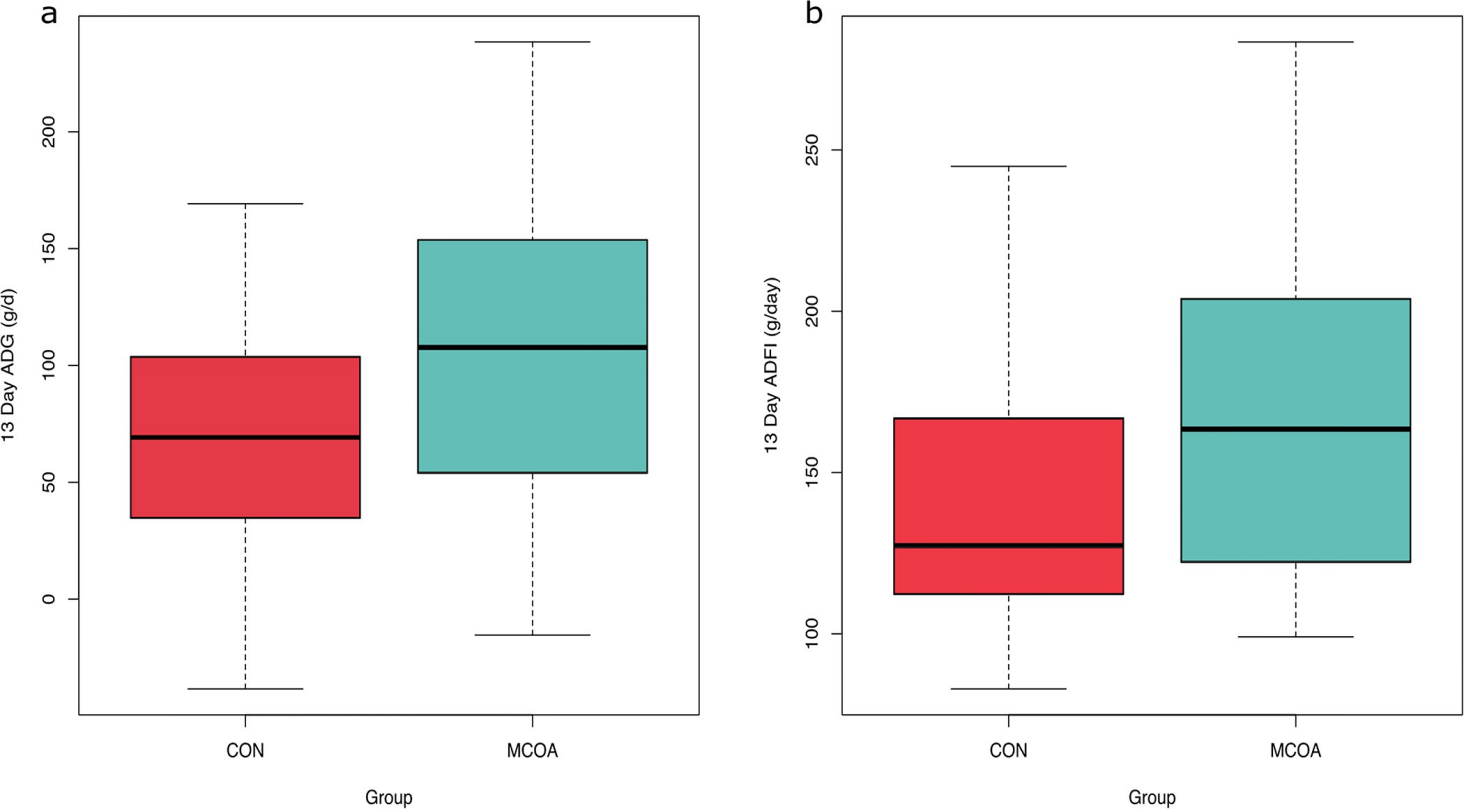
Comparison of growth performance. a. Average daily gain (ADG) and b. average daily feed intake (ADFI) for pigs fed a control diet (CON) or a control diet supplemented with blend of medium chain fatty acids and organic acids on top (MCOA) for the first thirteen days post-weaning. ADG was significantly different (*P* = 0.04) while ADFI was not significantly different (*P* = 0.19) between groups (n = 19 for CON and 22 for MCOA) for the thirteen-day period.

Average daily gain (ADG) from D0-13 (CON n = 19, MCOA n = 22) was higher for pigs fed MCOA (111 vs 71 g/d with *P* = 0.04; Fig 1), this growth rate is lower than expected for pigs of this age in this facility; likely due to the increased amount of handling required for this experimental protocol. Feed efficiency was not significantly different between groups.

### Metabolome

**Fig 2:**
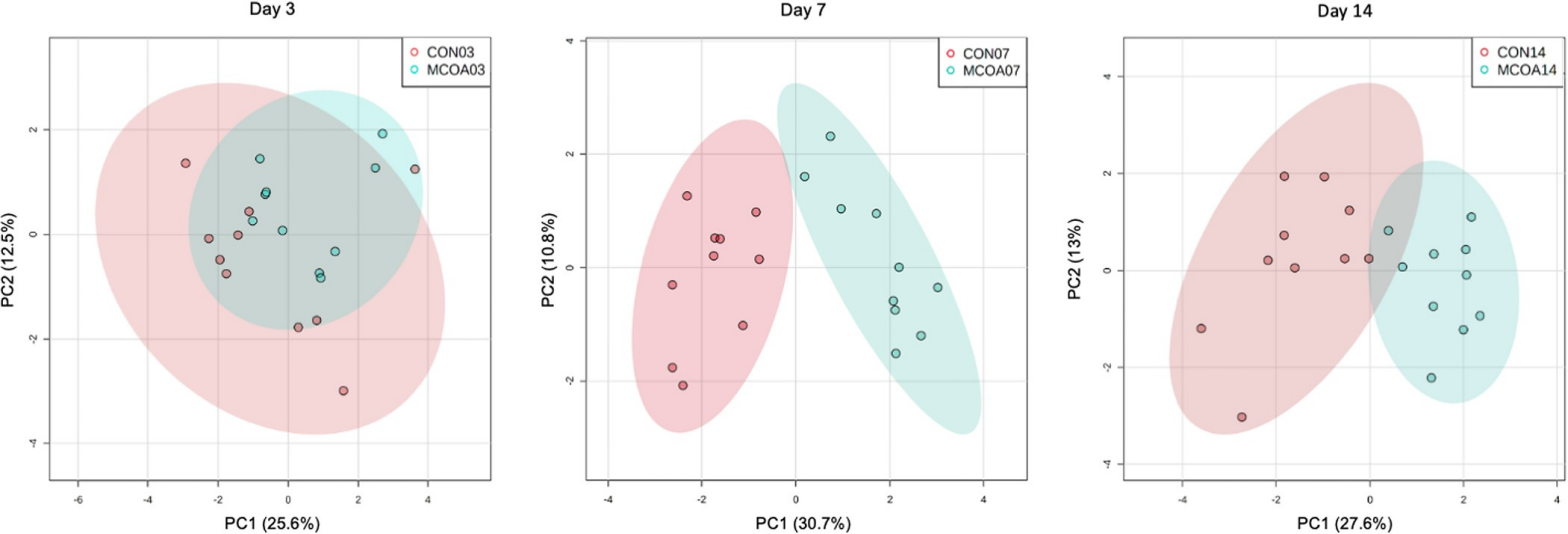
Summary of overall effects of MCOA on weaned pig metabolome. PCAs of metabolites on D3, 7, and 14 in jugular plasma; Group names indicate treatment (CON vs. MCOA) and day of sampling (7 or 14). Group names indicate treatment (CON vs. MCOA) and day of sampling (7 or 14).

**Fig 3:**
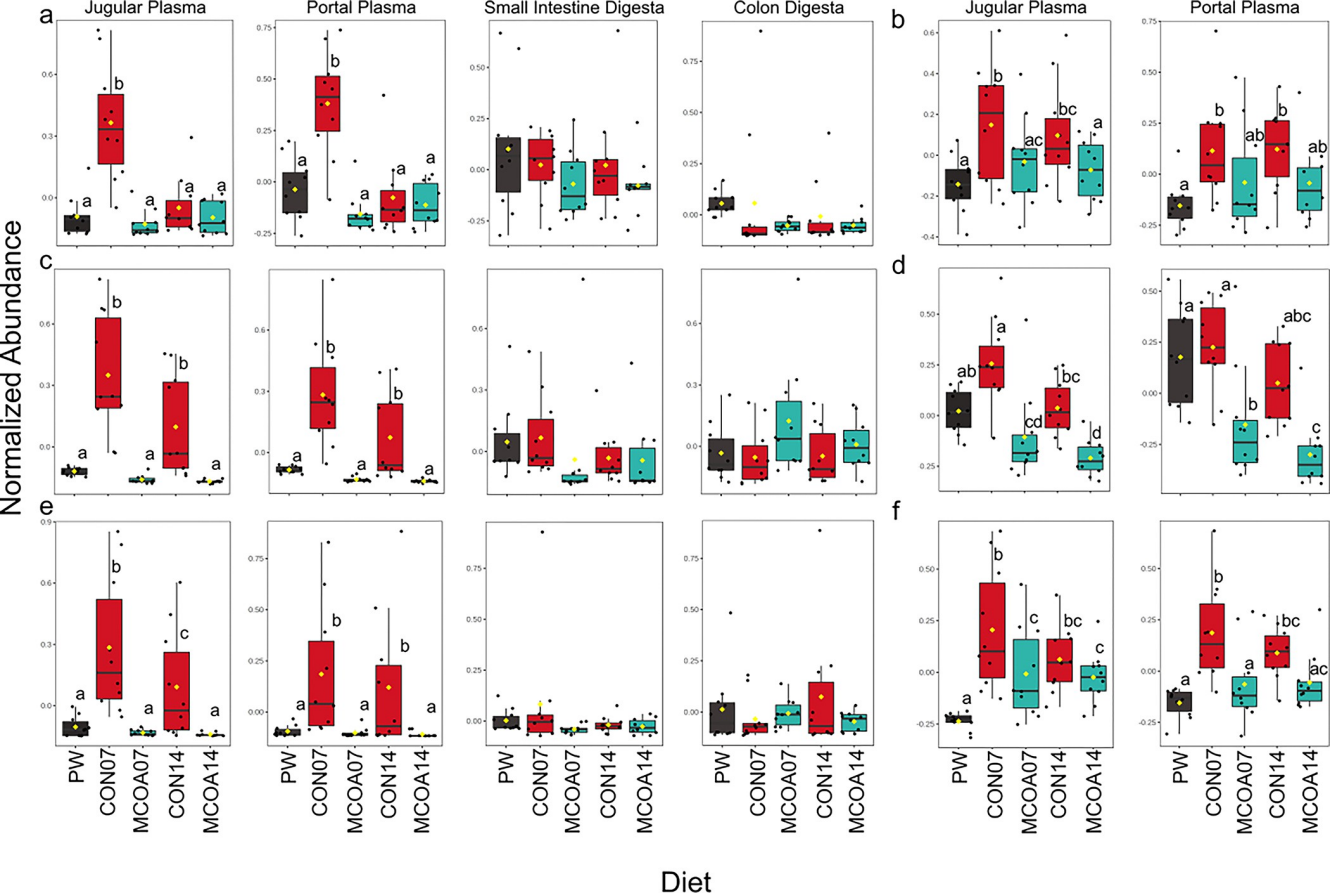
Whole-body metabolic indicators of tissue deposition. Differences between pre-weaning, MCOA, and control groups shown on D7 and 14 include a. Creatine; b. Creatinine; c. Acetylcarnitine; d. L-carnitine; e. ß-Hydroxybutyrate; f. Homocysteine. Metabolomics was performed using LC-MS/MS with a SWATH method for peak identification and analyzed in Metaboanalyst 5.0. Compounds shown in Fig 3B, 3D and 3F were not captured in intestinal LC-MS/MS. Group names indicate treatment (CON vs. MCOA) and day of sampling (7 or 14). Pre-weaning samples are denoted as PW. Groups with statistically different means (*P* < 0.05) are denoted by different letters.

**Fig 4:**
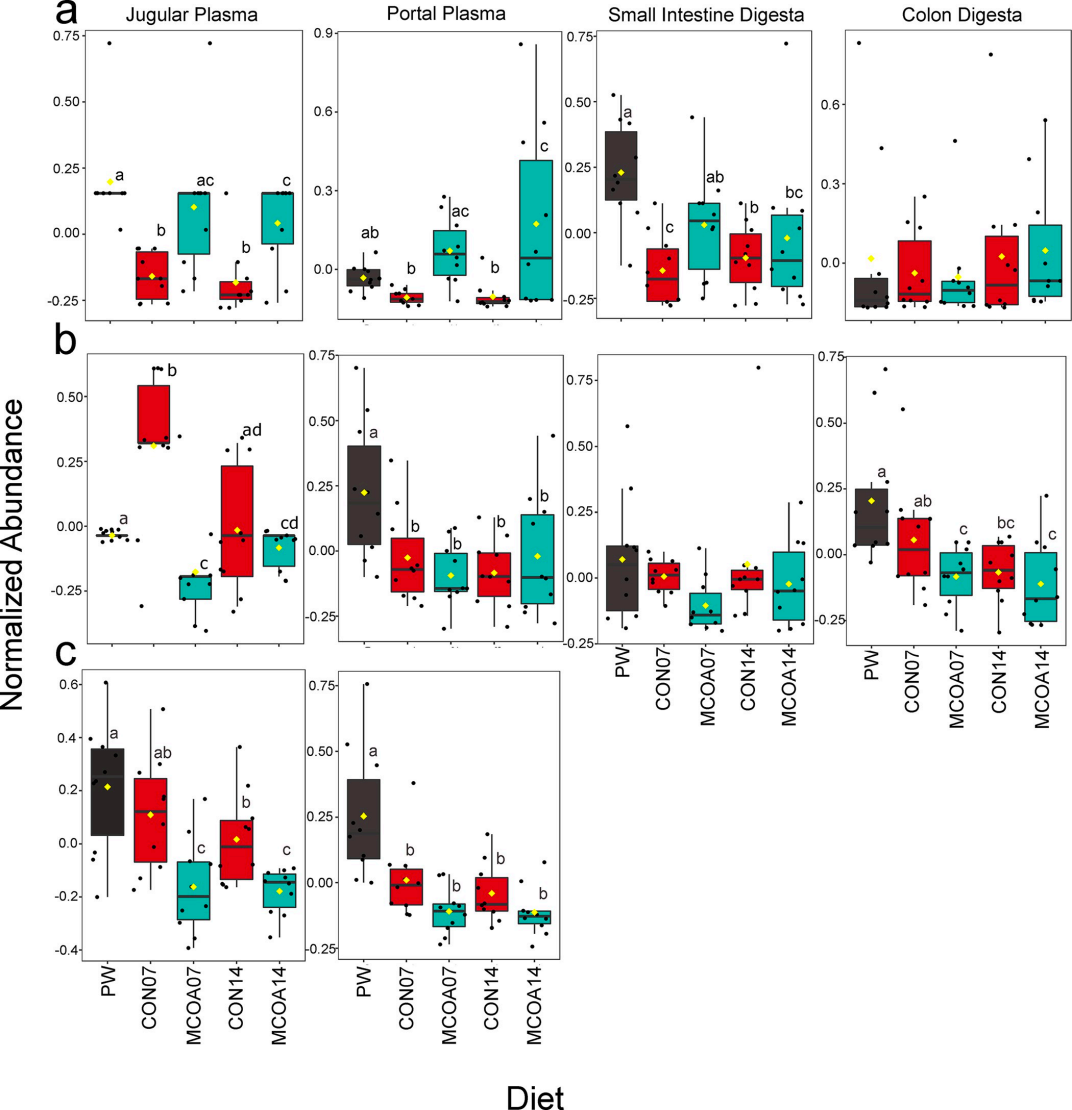
Bile acid changes observed on D7 and 14 post-weaning in response to feeding a control diet or a diet containing MCOA. a. Cholic acid; b. Choline; c. Taurine. Metabolomics was performed using LC-MS/MS with a SWATH method for peak identification and analyzed in Metaboanalyst 5.0. Taurine was not detected intestinal metabolome. Group names indicate treatment (CON vs. MCOA) and day of sampling (7 or 14). Pre-weaning samples are denoted as PW. Groups with statistically different means (*P* < 0.05) are denoted by different letters.

**Fig 5:**
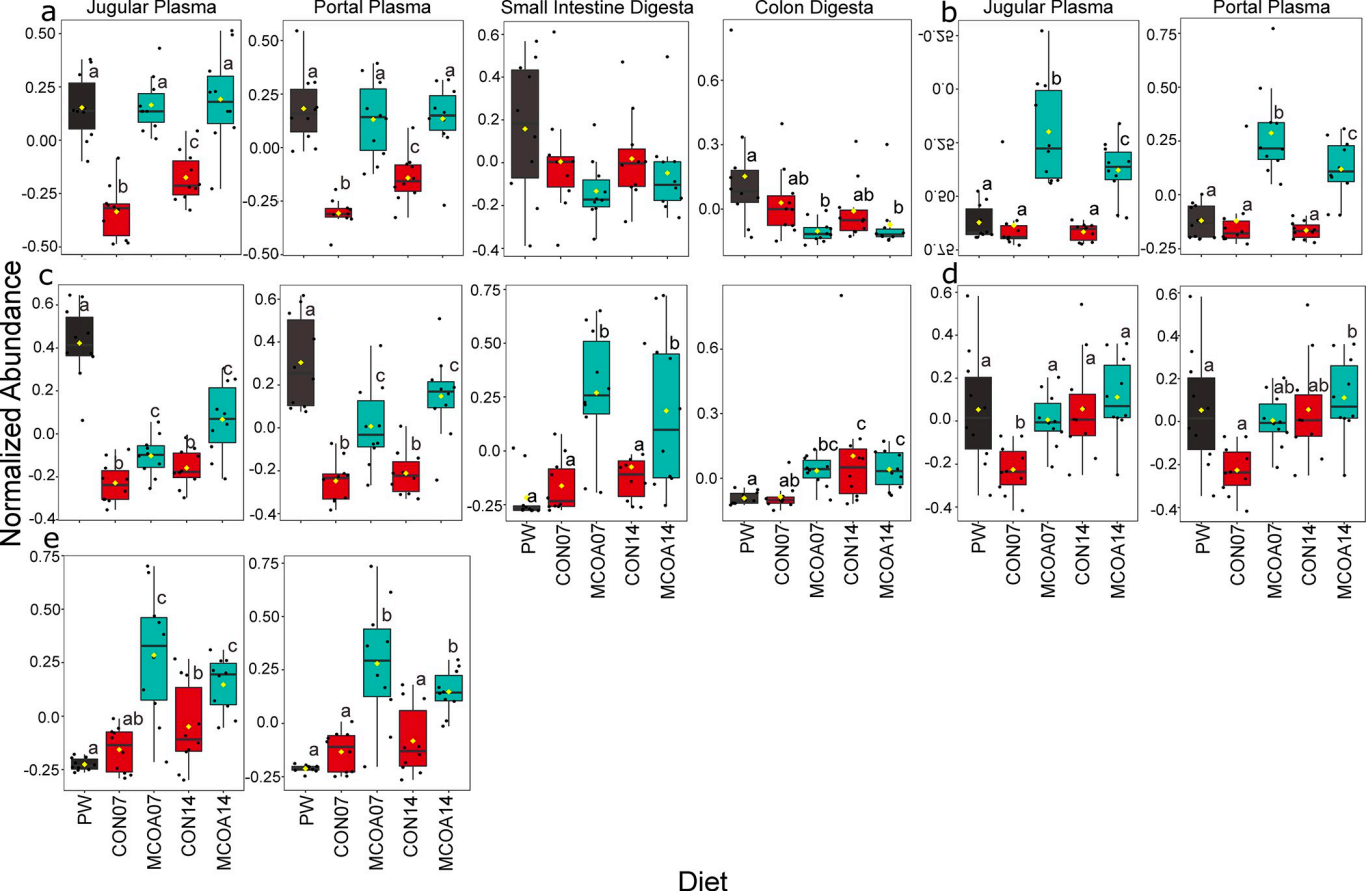
Tryptophan and metabolite alterations in response to a control diet or a diet containing MCOA. a Tryptophan; b. Indole-3-carboxylic acid; c. N-Acetyltryptophan; d. Indole-3-carboxyaldehyde; e. 3-Indolepropionic acid. Metabolomics analysis was performed using LC-MS/MS and a SWATH peak identification method. Statistical analysis was performed in Metaboanalyst 5.0. Compounds shown in 6b, d and e were not identified in intestinal metabolomics. Group names indicate treatment (CON vs. MCOA) and day of sampling (7 or 14). Pre-weaning samples are denoted as PW. Groups with statistically different means (*P* < 0.05) are denoted by different letters.

An overall shift in the global metabolite pool was observed at all time points, with the most noticeable difference at D7. Although feed intake was relatively low at D3 and 5 post-weaning in both diets (S1 Fig), analysis of similarity on Bray-Curtis distances showed a small but significant difference between plasma metabolome on both days (*P* = 0.004, r^2^ = 0.29 and *P* = 0.007, r^2^ = 0.29 respectively; Fig 2). No significant differences were detected in intestinal metabolites at D3 or 5. A more pronounced separation, with a greater number of significantly different metabolites was observed at D7 and 14 in jugular plasma (*P* = 0.001, r2 = 0.82 and *P* = 0.001, r^2^ = 0.70 respectively, Fig 2). When individual metabolites were compared, key metabolite groups with noted differences between MCOA and CON animals included markers of tissue deposition and fatty acid metabolism (Fig 3), bile acid components (Fig 4), tryptophan metabolites (Fig 5), and B vitamins (S2 Fig). Metabolic pathway results showed two significantly overrepresented pathways in MCOA pigs for tryptophan metabolism and riboflavin metabolism. In CON animals, three significantly overrepresented pathways for purine metabolism, betaine metabolism, and oxidation of branched-chain fatty acids were detected (S3 Fig). A trend for enriched beta oxidation of very long chain fatty acids and pyrimidine metabolism were also identified.

Differences in small intestinal metabolome were only detected on D7 post-weaning (*P* = 0.002, r2 = 0.27). In the colon, small but significant global differences were detected on D3 (*P* = 0.04, r2 = 0.10), while more pronounced changes were found on D7 and 14 (*P* = 0.001, r^2^ = 0.55 and *P* = 0.001, r^2^ = 0.30 respectively). The global differences observed in intestinal metabolome were not due to the residual presence of additive components in the digesta at either location. Differences in individual gastrointestinal metabolites, measured through FDR-adjusted t-tests, were only observed at D7.

### Intestinal microbiota

**Fig 6:**
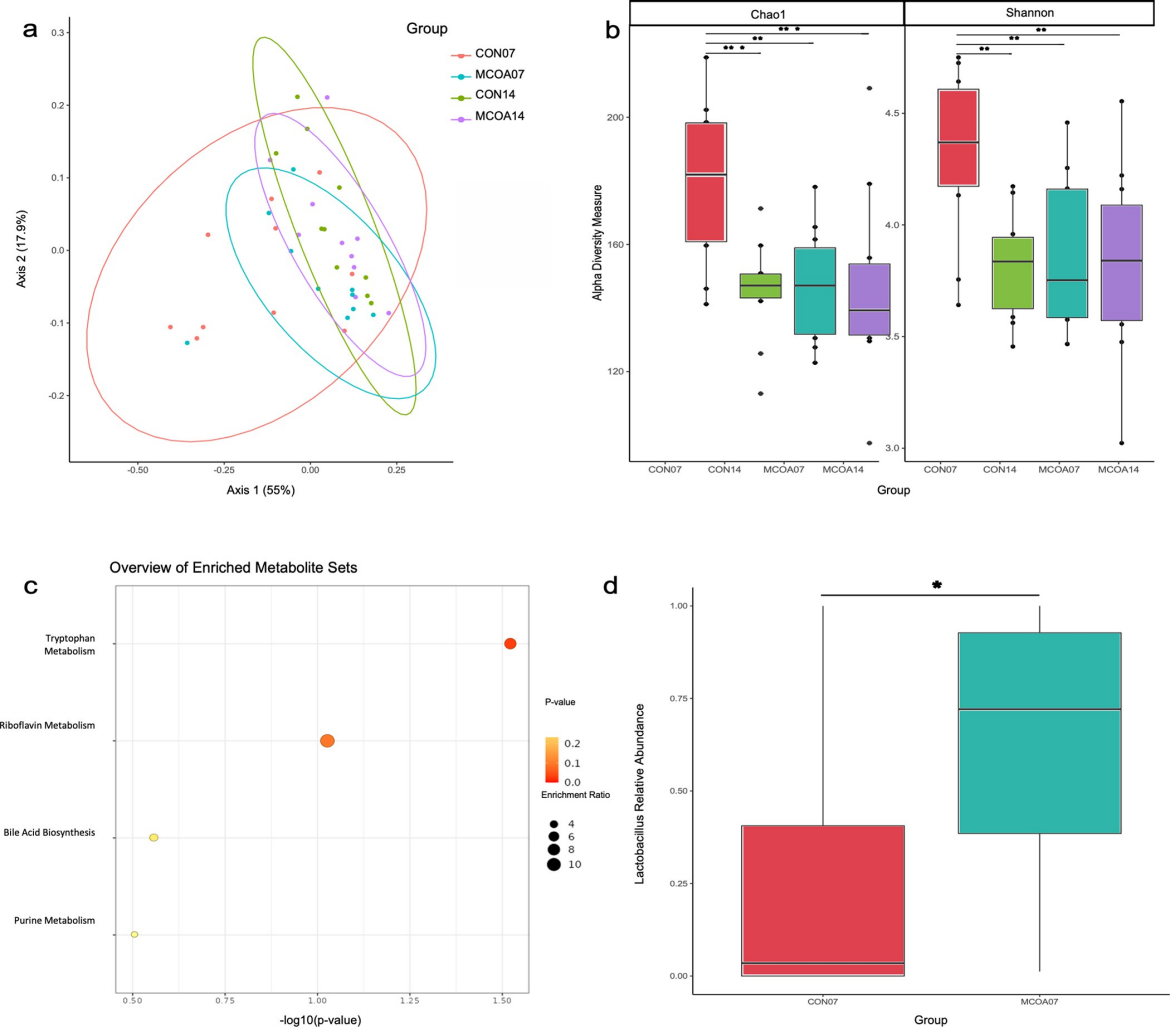
Summary of overall effects of MCOA on weaned pig microbiota and metabolic pathways. a. Weighted Unifrac distance of colon microbiota on D7 and 14; b. Alpha diversity measures of colon microbiota on D7 and 14; c. Metabolic pathways enriched in the jugular plasma of MCOA pigs on D7 post-weaning; d. *Lactobacillus* relative abundance D7 in the small intestine. Group names indicate treatment (CON vs. MCOA) and day of sampling (7 or 14). Differences are denoted as * P < 0.10, ** P < 0.05 and *** P < 0.01.

Analysis of relative abundance data from the small intestine at all time points revealed numerous low abundance opportunistic taxa in the small intestinal microbiota at D3, 5 and 7, which limited statistical comparisons of important native microbiota constituents at early timepoints. To overcome this interpretation challenge in the early post-weaning period, a successional core of microbes was identified using D14 sequencing results. The abundance of these core taxa was then examined at earlier timepoints to measure microbial succession during this period. At D14, a total of four taxa comprising 83–100% of the small intestinal microbiota were identified as core genera: *Clostridium sensu*; Escherichia_Shigella, *Lactobacillus* and *Streptococcus*. Comparison of these taxa between MCOA and CON fed animals revealed a trend toward increased *Lactobacillus* relative abundance at D7 post-weaning in MCOA fed pigs (*P* = 0.05) (Fig 6D). This re-establishment of *Lactobacillus* is shown in the small intestinal microbiota of MCOA but not CON fed pigs at D7 and coincides with the time point where the most pronounced differences in metabolome in all compartments was observed.

Analysis of colon microbiota showed differences in composition and alpha diversity metrics between treatment groups at D7 post-weaning. The microbiota of MCOA fed pigs at D7 tended to be different than that of CON fed pigs (*P* = 0.09), and more closely resembled the microbiota of pigs at D14 post-weaning in measures of both overall abundance and composition (Fig 6A and 6B). The variability of individual microbiota composition, measured by homogeneity of dispersion, in CON fed animals at D7 was quite high (Fig 6A) compared to those at D14 (CON07 vs. CON14, *P* = 0.001), while that of MCOA fed animals was not significantly different from D14 animals (MCOA07 vs. MCOA14, *P* = 0.13).

### Microbiota and metabolome networks

**Fig 7:**
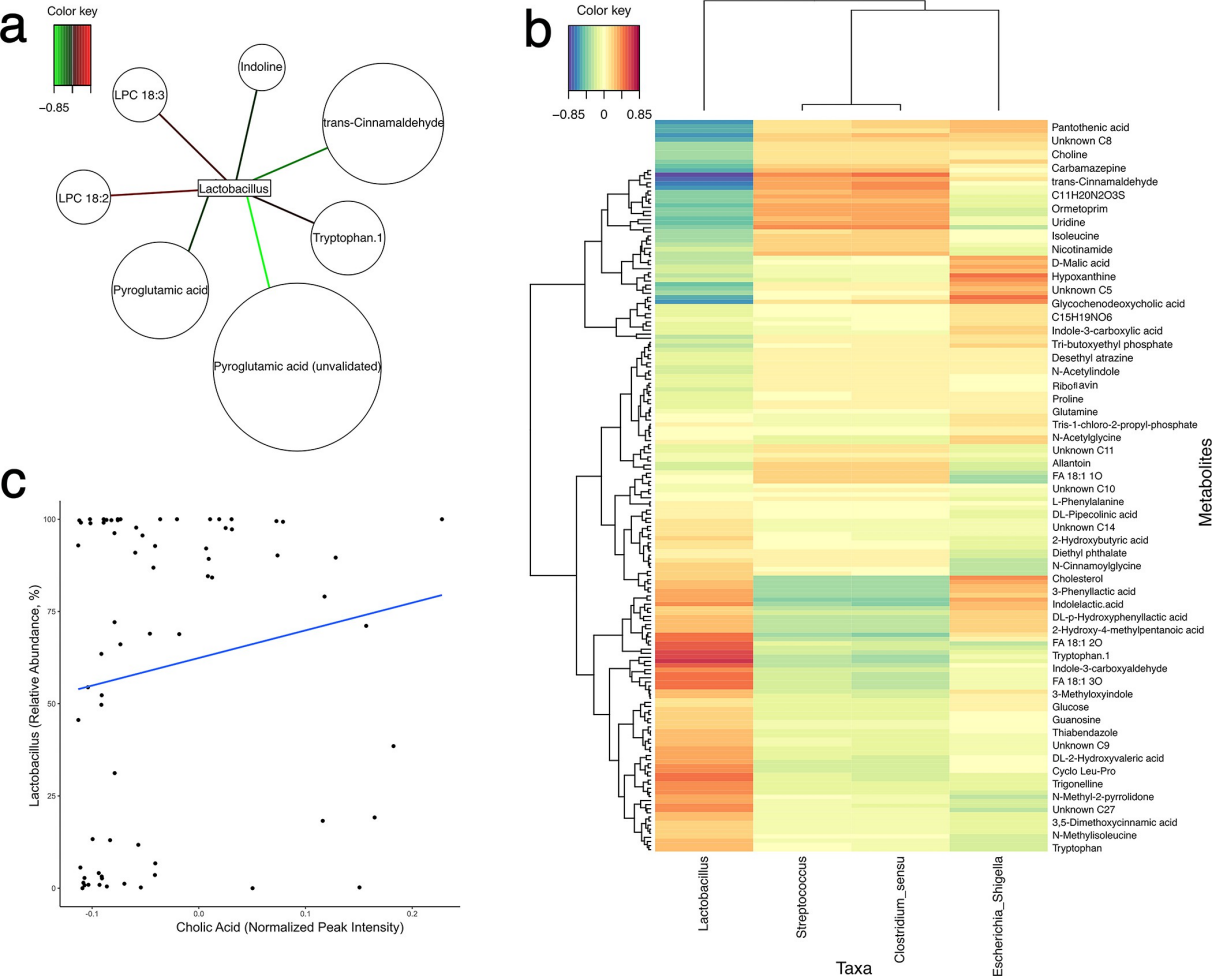
Microbe-metabolite relationships. a. Network analysis of core SI intestinal microbiota and metabolites at D7 with a significant correlation (*P* < 0.05) threshold r^2^ of 0.6; b. Microbe-metabolite network analysis results for all significant correlations (*P* < 0.05) with core small intestine microbial taxa on D7; c. Correlation between *Lactobacillus* relative abundance and cholic acid in all pigs from the study (*P* = 0.08, rho = 0.21).

Network analysis using the R package mixOmics [43] revealed a positive correlation between *Lactobacillus* and abundance of tryptophan (*P* < 0.05, r^2^ = 0.61) and tryptophan metabolites (*P* < 0.05, r^2^ = 0.40–0.62) found in jugular plasma at D7 post-weaning(Fig 7A and 7B).

As *Lactobacillus* species have bile-tolerance mechanisms [44], the relationship between cholic acid and *Lactobacillus*, both of which are more abundant in MCOA fed pigs at D7, was explored in pigs sampled at all timepoints using Spearman correlation. A trend for a positive correlation was observed (*P* = 0.08, rho = 0.21; Fig 7C).

## Discussion

### Patterns of metabolome differences

The overall pattern of metabolite changes, with the strongest difference between metabolomes observed at D7 post-weaning, is particularly relevant given that it coincides with a time when the risk of *E*. *coli* associated post-weaning diarrhea is high and impacts of post-weaning interventions may be important for preventing diarrheal disease [45]. This is also a time where the initial acute stressors of weaning are resolved, and feed intake begins to rebound [45]. Higher feed intake at D7 and 14 would result in higher MCOA intake, contributing to the global differences observed on these days as well as to the potential mechanism underlying the global difference in intestinal metabolome observed at D7.

The fact that differences observed in the intestinal metabolite profiles did not include the additive components themselves is not unexpected as previous studies in weanling pigs have shown most MCFA digestion and absorption occurs prior to the end of the proximal small intestine, and abundance of these compounds, except for slow-release C12, drops sharply in subsequent sections of the GI tract [16, 46]. Consistent with our results, it is expected that these compounds would not be observed in total small intestine or colon contents. However, this result alongside the changes in individual metabolites observed demonstrate the role of MCOA in altering metabolic networks beyond the metabolism of additive residues.

### Metabolites reflective of impacts on tissue deposition

Several notable differences in metabolic markers of fatty acid metabolism and tissue deposition were detected in jugular plasma at D7 and 14 post-weaning. The prevailing pattern suggests improved energy balance and muscle metabolism alongside decreased fatty acid catabolism in MCOA fed pigs. Key evidence supporting this is the pattern of decreased creatine, creatinine and β-hydroxybutyrate observed in MCOA fed pigs at these time points (Fig 3A, 3B and 3E). At D7 post-weaning, decreased acetylcarnitine, L-carnitine, and homocysteine also support this finding (Fig 3C, 3D and 3F). Except for homocysteine and creatine, these differences were also observed on D14 indicating ongoing effects of MCOA in increasing tissue metabolism post-weaning. Carnitine abundance is an important indicator of fatty acid metabolism and for overall metabolic status due to its effects on lipid and glucose homeostasis [47, 48]. L-carnitine levels are tightly regulated in response to whole-body nutrient sensing, matching the amount of fatty acid metabolism to what is needed to regulate metabolic status [48]. The lower levels of L-carnitine in MCOA fed pigs may indicate less tissue catabolism at D7 and 14 post-weaning. At the same timepoints, lower circulating creatinine observed in MCOA fed pigs (Fig 3A), also suggests higher muscle metabolism. This is further supported by the higher growth observed from D0-13 of pigs fed MCOA. There was a dramatic increase in creatine post-weaning in CON fed pigs, consistent with reduced feed intake alongside a corresponding reduction in metabolic activity [49], while MCOA fed pigs did not exhibit this increase. Lower levels of creatinine (Fig 3B) are also seen in MCOA fed pigs, while CON fed pigs display elevated creatinine on D7 and 14 further indicating decreased metabolic activity and negative energy balance in CON fed pigs post-weaning [50, 51]. This is further supported by significant overrepresentation of metabolites in pathways for oxidation of fatty acids (S3 Fig). Higher levels of riboflavin observed in plasma at D7 and 14 in MCOA fed pigs (S2 Fig) may also be related to these changes in energy balance. Levels of the B-vitamins niacin, riboflavin, and folate may also reflect creatine synthesis as they provide required methyl groups [52]. During fasting, methyl requirements for the methionine cycle must be met through remethylation of homocysteine, which depends on B-vitamin availability [53]. This results in an inverse relationship between homocysteine, creatinine and B-vitamin metabolism and status [54], as observed here. At D7 post-weaning, riboflavin metabolites were found to be significantly enriched in jugular plasma of MCOA fed pigs (Fig 6C), while nicotinamide and pantothenic acid did not show the same pattern (S2 Fig). β-hydroxybutyrate was also elevated in CON fed pigs at D7 and 14 post-weaning (Fig 3E), indicating ongoing negative energy balance in CON fed pigs [55], despite comparable feed intake between groups (Fig 1B). The differences in these metabolites taken together illustrate decreased body fat mobilization in MCOA fed pigs at D7 and 14 post-weaning, which could in part be due to improved nutrient digestion, absorption, and subsequent alterations in nutrient sensing in the gastrointestinal tract in the presence of MCOA [56].

### MCOA supplementation alters bile components

At D7 post-weaning, MCOA fed pigs had increased abundance of cholic acid in the portal plasma, jugular plasma, and colon, suggesting an increase in bile acid production and secretion (Fig 4A). This change was accompanied by a decrease in the abundance of the bile acid precursor taurine in portal and jugular plasma (Fig 4B and 4C). Taurine also has an important role in osmoregulation [57], and may therefore be associated with post-weaning edema. Decreased plasma choline in MCOA pigs may also be due to changes in bile production and secretion, as it is the precursor to phosphatidylcholine, the primary phospholipid in bile as well as an important regulator of cholesterol homeostasis [58, 59]. This alteration in primary bile metabolites persisted at D14 post-weaning, showing an effect of MCOA on bile metabolism throughout the sensitive post-weaning period as shown in Fig 4. Medium-chain fatty acids are known to increase the production of primary bile acids compared to other dietary sources of fat [60]. This could be particularly advantageous in the post-weaning period where bile acid and lipase production are inadequate [5, 61, 62], and decreased feed intake may alter signaling for bile acid production, resulting in decreased nutrient digestibility [63, 64].

### MCOA supplementation increases generation of bioactive indoles

Alongside changes in bile metabolites, pigs fed MCOA displayed higher concentrations of tryptophan at D7 post-weaning in the portal and jugular plasma, while a lower concentration was detected in the colon (Fig 5A). At the same time-point, higher levels of five tryptophan metabolites–indole-3-carboxylic acid, 3-methyloxyindole, 2-methyloxyindole, indole-3-carboxaldehyde and 3-indolepropionic acid (IPA) were detected in the plasma of pigs receiving MCOA on D7 (Fig 5B, 5D and 5E; S4 Fig). Overrepresentation analysis further supported a significant increase in the tryptophan metabolism in MCOA-fed pigs on D7 post-weaning (Fig 4C). Increases in IPA, indole-3-carboxylic acid, 3-methyloxyindole, and 2-methyloxyindole were also observed in plasma at D14 (Fig 5B, 5D and 5E; S4 Fig). Indole and its derivatives can be absorbed via passive diffusion through cell membranes and readily move into portal circulation [65], and it is, therefore, not surprising that these metabolites did not differ in the digesta. Further, an increase in n-acetylated tryptophan, a microbial product [66], was detected in the small intestine of MCOA fed pigs (Fig 5C). These changes are indicative of an increase in tryptophan degradation by gastrointestinal microbes in MCOA fed pigs. As tryptophan metabolites are important signaling molecules [67, 68], changes in tryptophan availability and production of bioactive molecules via microbial degradation are of particular interest to animal health outcomes. These tryptophan metabolites are commonly detected in a mature pig microbiota [69], and are therefore of interest for further investigation related to post-weaning microbiota assembly in this study.

Accumulating evidence shows that indoles are highly bioactive through aryl-hydrocarbon receptor (AhR) binding and are important regulators of gut barrier function with protective effects in models of irritable bowel disease [70–72]. Serum indole and IPA have previously been shown to be decreased in active colitis, while colonic tryptophan was elevated [69]. This supports a positive effect of MCOA on gastrointestinal tract function and inflammation as the opposite pattern is observed at a time where gastrointestinal inflammation is common [1, 73]. Indole-3-carboxylic acid is also capable of binding AhR, though this has been less widely described [74]. Methylindole (skatole) is an intermediate in the production of indole-3-aldehyde from indole-3-acetate and is subsequently metabolized into 3-methyloxyindole in the liver via CYP450 [65]. Though not likely to be related to weaning outcomes, this metabolite may be of further interest for minimizing off-flavours in pork products.

### Microbiota assembly differences in response to MCOA

Given the role of *Lactobacillus* spp. as important members of the porcine microbiota [75], which are long known to be sensitive to host stressors [76], their increased abundance in MCOA-fed pigs is an important indicator of microbiota re-assembly following weaning [77]. The positive correlation between this genus and cholic acid is of further interest, indicating that the inclusion of MCOA and resulting increase in cholic acid production may shape the small intestinal microbiota’s re-assembly of *Lactobacillus* post-weaning (Fig 7C). Further supporting this is the consistent evidence in the literature of increased lactobacilli counts in a variety of age groups of pigs in response to a blend of dietary MCOA [28, 29, 78, 79], suggesting that a protective effect of MCOA occurs through shaping a gut environment that allows an earlier *Lactobacillus* expansion post-weaning.

In the early post-weaning period, alterations in tryptophan and indole metabolites between MCOA and CON fed pigs suggests that MCOA may increase the assembly of microbes able to generate indole metabolites [80]. Production of IPA and the correlation with *Lactobacillus* abundance, may be of particular interest due to its beneficial effects on gut barrier function [70], requires microbial enzymatic machinery, and common commensal gut organisms *Lactobacillus*, *C*. *sporogenes* and *Peptostreptococci* possess the capability to generate IPA [65, 80, 81]. Encapsulated MCFAs have been shown to increase *Lactobacillus* spp. abundance in the stomach of pigs [16], supporting the possibility that tryptophan degradation by lactobacilli could begin in the proximal gastrointestinal tract in MCOA fed pigs and result in the observed increase in acetylated tryptophan in the small intestine of MCOA fed pigs.

The tendency of MCOA-fed pigs at D7 to have a colonic microbiota that was different than that of CON pigs, and similar to that of D14 samples, demonstrates that MCOA supports a more expedient assembly of the post-weaning colonic microbiota, resulting in a more uniform microbiota composition with fewer incidental taxa observed. This timely reassembly of the microbiota in both the small intestine and colon at D7 post-weaning is a potential benefit of MCOA, which will allow pigs to move through the vulnerable weaning period. The weaning transition period is a critical time where gut microbiota disruption can facilitate opportunistic pathogen growth resulting in post-weaning diarrhea [1, 15]. There is an opportunity to improve animal health and growth outcomes during this sensitive window by assisting the pig with efficient assembly of a microbiome that is adapted to the post-weaning diet, thereby supporting pathogen exclusion, and promoting optimal immune function [10, 11, 82]. Improved understanding of the mechanisms by which MCOA modulate the gut microbiota can contribute to the successful application of this additive after weaning.

## Conclusion

In this paper we describe the effects of a blend of medium chain fatty acids, organic acids, slow release C12, target release butyrate and a phenolic compound on microbes, metabolites, and their interactions. The effect of MCOA on bile acid dynamics offers novel insights into the mechanism by which these additives might confer benefits during the post-weaning period. The correlation between cholic acid and *Lactobacillus* relative abundance provides a possible mechanism underlying this consistently observed effect. Lastly, the relationship between *Lactobacillus* in the small intestine and tryptophan metabolism presents a new understanding of the generation of indole species in the porcine gastrointestinal tract. While these metabolites are abundant in the metabolome of pigs, it is not known where microbial tryptophan metabolism begins. Finally, the relationship between MCOA, bile acid production and tissue catabolism provides an interesting area for further exploration in the weaning transition. Bile acid secretion is typically reduced post-weaning, and the effect of MCOA could allow the post-weaning pig to maintain its ability to digest and absorb lipids and, thus, protect against post-weaning catabolism. This is an interesting avenue for further research into the relationship between MCOA, and whole-body metabolism in the weaned pig.

## Supporting information

S1 Fig Comparison of daily feed intake of pigs from Day 3–7 post-weaning for pigs fed a control diet (CON) or a control diet supplemented with blend of medium chain fatty acids and organic acids on top (MCOA). Group names indicate treatment and day of sampling (7 or 14).
(TIFF)

S2 Fig Differences in b-vitamins in response to a control diet or a diet containing MCOA: a. riboflavin, b. nicotinamide, c. pantothenic acid. Metabolomics analysis was performed using LC-MS/MS and a SWATH peak identification method. Statistical analysis was performed in Metaboanalyst 5.0. Compounds shown were not identified in intestinal metabolomics. Group names indicate treatment (CON vs. MCOA) and day of sampling (7 or 14). Pre-weaning samples are denoted as PW. Groups with statistically different means (*P* < 0.05) are denoted by different letters.
(TIFF)

S3 Fig Metabolite pathways enriched in jugular plasma of CON pigs on D7 post-weaning. (TIFF)

S4 Fig Additional tryptophan metabolite alterations in response to a control diet or a diet containing MCOA: a. 3-methyloxyindole, b. 2-methyloxyindole. Metabolomics analysis was performed using LC-MS/MS and a SWATH peak identification method. Statistical analysis was performed in Metaboanalyst 5.0. Compounds shown were not identified in intestinal metabolomics. Group names indicate treatment (CON vs. MCOA) and day of sampling (7 or 14). Pre-weaning samples are denoted as PW. Groups with statistically different means (*P* < 0.05) are denoted by different letters.
(TIFF)

## Abbreviations

AhR: aryl hydrocarbon receptor
IPA: indole-3-propionic Acid
MCFAs: medium-chain fatty acids
rCCA: regularised Canonical Correlation Analysis
