# Supplementary figures and images for: A blend of medium-chain fatty acids, butyrate, organic acids, and a phenolic compound accelerates microbial maturation in newly weaned piglets
Source: PLoS One. 2023 Jul 28;18(7):e0289214. doi: 10.1371/journal.pone.0289214 (PMC10381057; doi:10.1371/journal.pone.0289214)

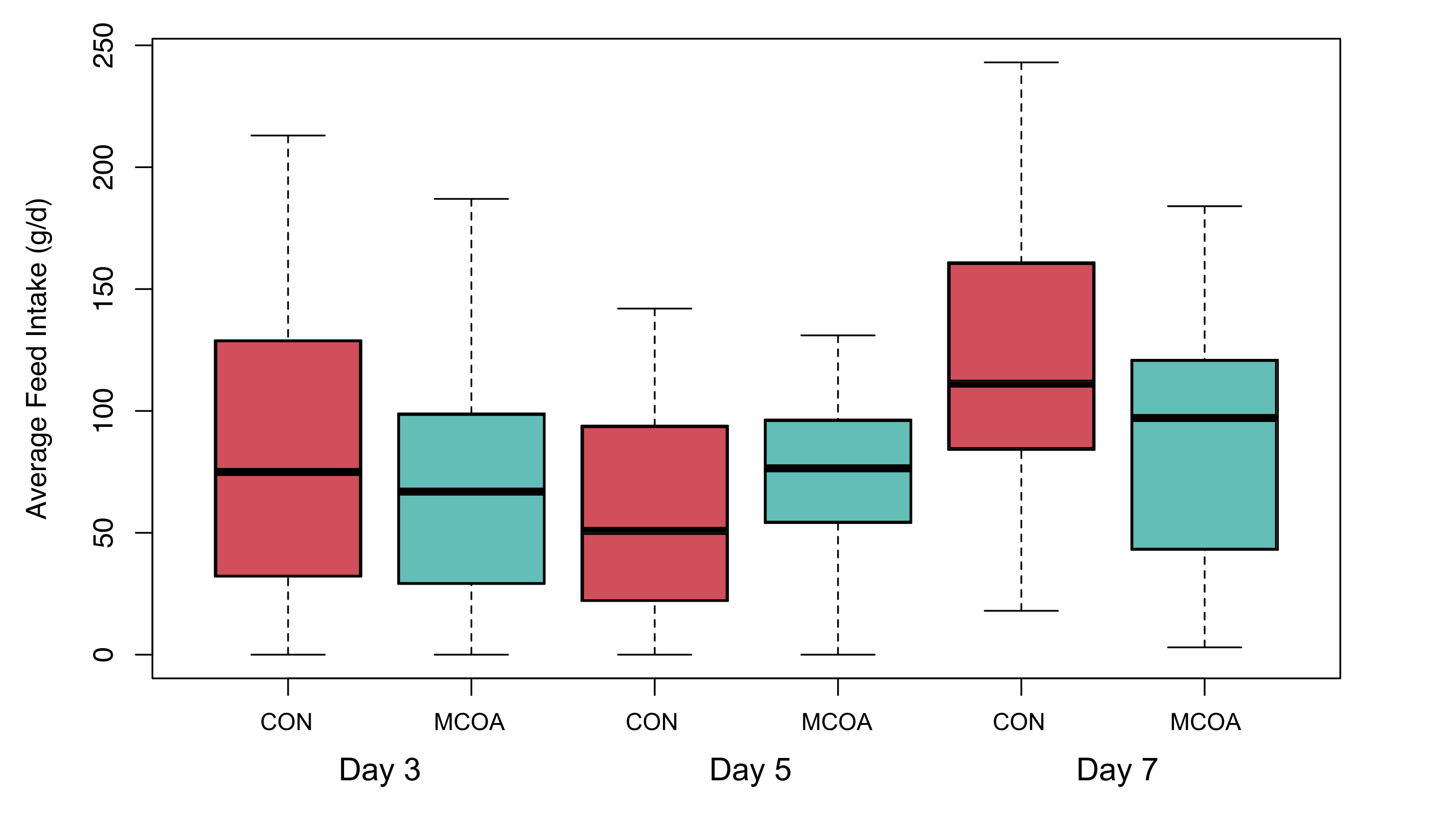

Supplement: S1 Fig — Group names indicate treatment and day of sampling (7 or 14). (TIFF) [file pone.0289214.s001.tiff]

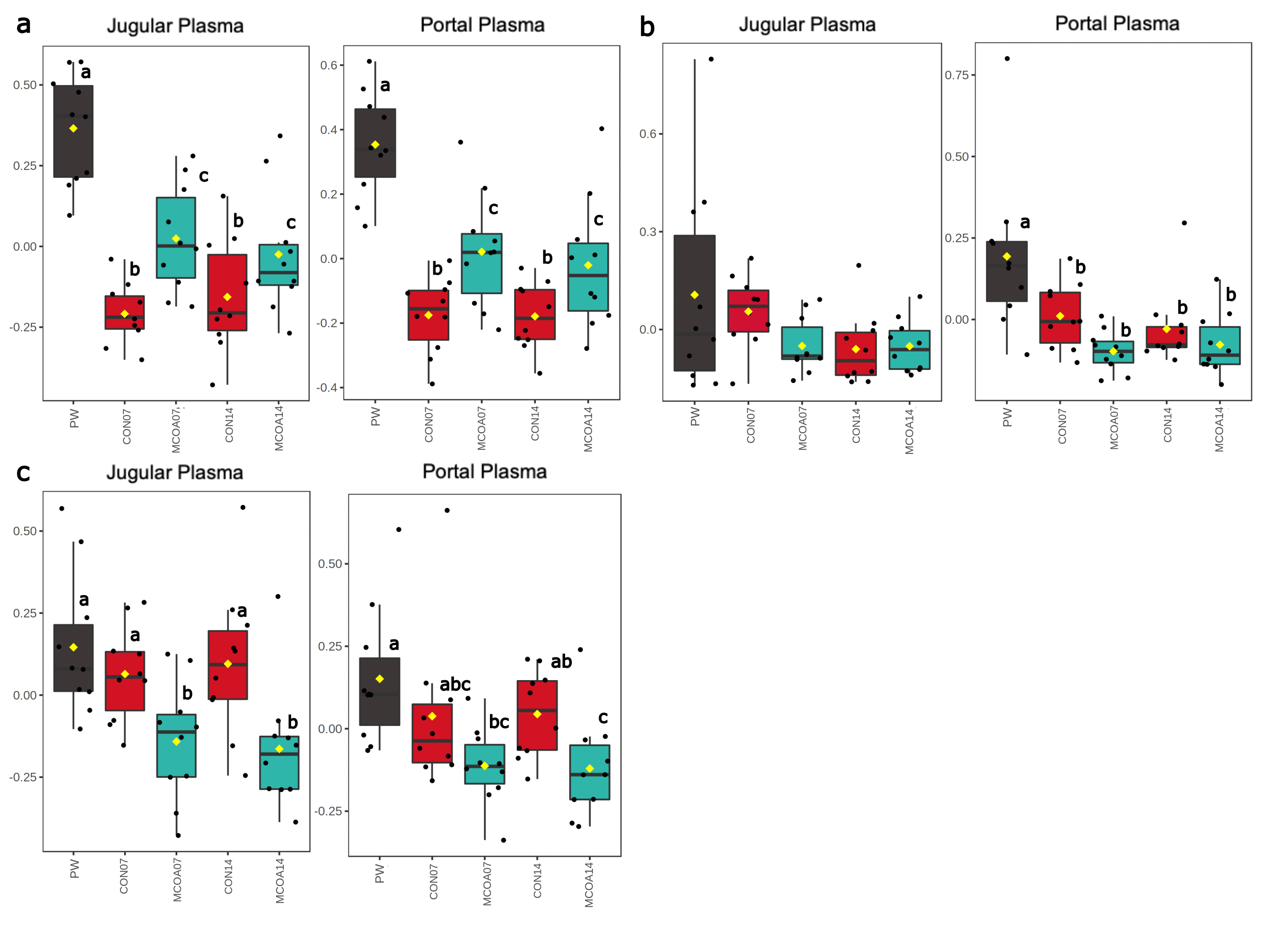

Supplement: S2 Fig — riboflavin, b. nicotinamide, c. pantothenic acid. Metabolomics analysis was performed using LC-MS/MS and a SWATH peak identification method. Statistical analysis was performed in Metaboanalyst 5.0. Compounds shown were not identified in intestinal metabolomics. Group names indicate treatment (CON vs. MCOA) and day of sampling (7 or 14). Pre-weaning samples are denoted as PW. Groups with statistically different means (P < 0.05) are denoted by different letters. (TIFF) [file pone.0289214.s002.tiff]

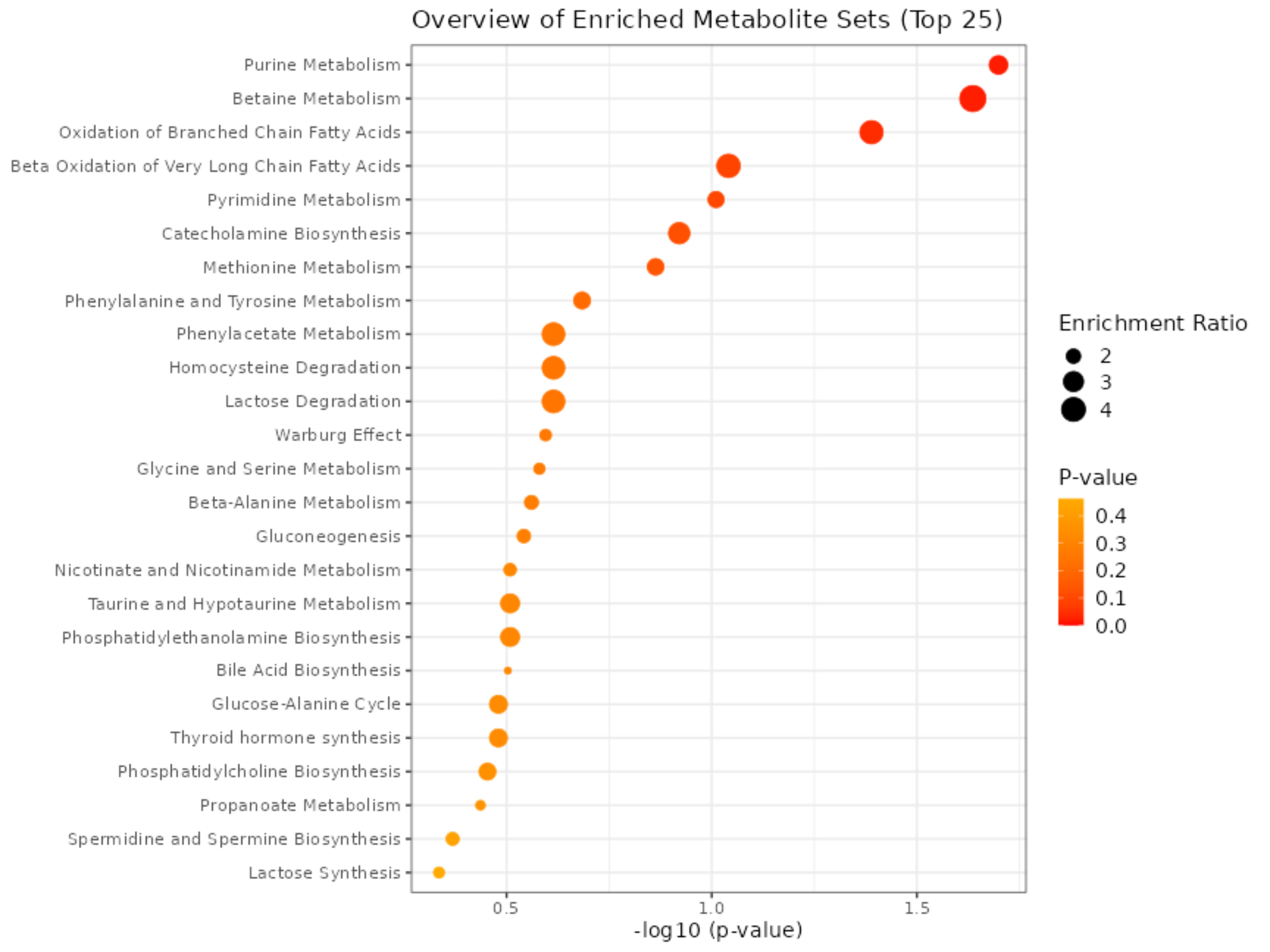

Supplement: S3 Fig — (TIFF) [file pone.0289214.s003.tiff]

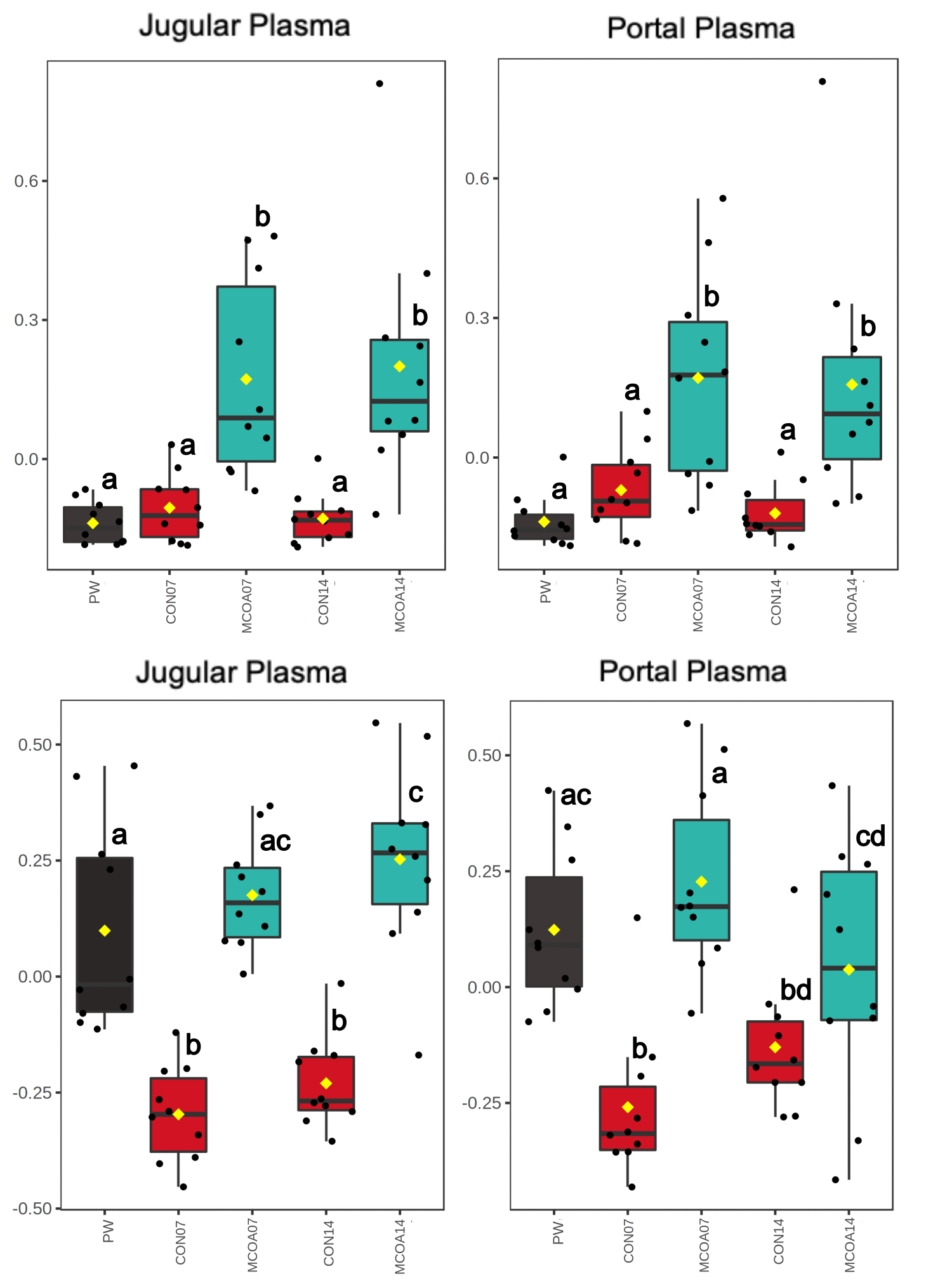

Supplement: S4 Fig — 3-methyloxyindole, b. 2-methyloxyindole. Metabolomics analysis was performed using LC-MS/MS and a SWATH peak identification method. Statistical analysis was performed in Metaboanalyst 5.0. Compounds shown were not identified in intestinal metabolomics. Group names indicate treatment (CON vs. MCOA) and day of sampling (7 or 14). Pre-weaning samples are denoted as PW. Groups with statistically different means (P < 0.05) are denoted by different letters. (TIFF) [file pone.0289214.s004.tiff]
